# Interplay of Glycemic Index, Glycemic Load, and Dietary Antioxidant Capacity with Insulin Resistance in Subjects with a Cardiometabolic Risk Profile

**DOI:** 10.3390/ijms19113662

**Published:** 2018-11-20

**Authors:** Cristina Galarregui, María Ángeles Zulet, Irene Cantero, Bertha Araceli Marín-Alejandre, José Ignacio Monreal, Mariana Elorz, Alberto Benito-Boillos, José Ignacio Herrero, Josep Antoni Tur, Itziar Abete, José Alfredo Martínez

**Affiliations:** 1 Department of Nutrition, Food Science and Physiology and Centre for Nutrition Research, University of Navarra, 31008 Pamplona, Spain; cgalarregui@alumni.unav.es (C.G.); mazulet@unav.es (M.Á.Z.); icantero.1@alumni.unav.es (I.C.); bmarin.1@alumni.unav.es (B.A.M.-A.); jalfmtz@unav.es (J.A.M.); 2 Biomedical Research Centre Network in Physiopathology of Obesity and Nutrition (CIBERobn), Instituto de Salud Carlos III (ISCIII), 28029 Madrid, Spain; pep.tur@uib.es; 3 Navarra Institute for Health Research (IdiSNA), 31008 Pamplona, Spain; jimonreal@unav.es (J.I.M.); marelorz@unav.es (M.E.); albenitob@unav.es (A.B.-B.); iherrero@unav.es (J.I.H.); 4 Clinical Chemistry Department, University Clinic of Navarra, University of Navarra, 31008 Pamplona, Spain; 5 Radiodiagnostic Department, University Clinic of Navarra, University of Navarra, 31008 Pamplona, Spain; 6 Liver Unit, University Clinic of Navarra, University of Navarra, 31008 Pamplona, Spain; 7 Research Group on Community Nutrition and Oxidative Stress, Universitat de les Illes Balears, E-07122 Palma, Spain; 8 Precision Nutrition and Cardiometabolic Health Department, Madrid Institute of Advanced Studies (IMDEA Food), 28049 Madrid, Spain

**Keywords:** antioxidants, glucose, diet, diabetes, precision nutrition

## Abstract

Background: Dietary total antioxidant capacity (TAC), glycemic index (GI), and glycemic load (GL) are accepted indicators of diet quality, which have an effect on diet–disease relationships. The aim of this study was to evaluate potential associations of dietary TAC, GI, and GL with variables related to nutritive status and insulin resistance (IR) risk in cardiometabolic subjects. Methods: A total of 112 overweight or obese adults (age: 50.8 ± 9 years old) were included in the trial. Dietary intake was assessed by a validated 137-item food frequency questionnaire (FFQ), which was also used to calculate the dietary TAC, GI, and GL. Anthropometrics, blood pressure, body composition by dual-energy X-ray absorptiometry (DXA), glycemic and lipid profiles, C-reactive protein (CRP), as well as fatty liver quantification by magnetic resonance imaging (MRI) were assessed. Results: Subjects with higher values of TAC had significantly lower circulating insulin concentration and homeostatic model assessment of insulin resistance (HOMA-IR). Participants with higher values of HOMA-IR showed significantly higher GI and GL. Correlation analyses showed relevant inverse associations of GI and GL with TAC. A regression model evidenced a relationship of HOMA-IR with TAC, GI, and GL. Conclusion: This data reinforces the concept that dietary TAC, GI, and GL are potential markers of diet quality, which have an impact on the susceptible population with a cardiometabolic risk profile.

## 1. Introduction

Oxidative stress (OS) is recognized as a contributor to the pathogenesis of obesity, type 2 diabetes, atherosclerosis, and nonalcoholic fatty liver disease (NAFLD) among others [1]. Interestingly, OS, which is defined as a disturbance in the balance between the production of reactive oxygen species (free radicals) and the antioxidant defense system in body cells [2], has been related to insulin resistance (IR), a pathological condition where a normal or elevated insulin level produces an attenuated biological response [3].

The interplay between nutrition and metabolic status has important health implications. Indeed, dietary antioxidant intake is being considered as a protective factor against cell oxidative damage and related metabolic complications [4]. Moreover, it has been suggested that a dietary pattern characterized by high antioxidant capacity could be inversely related to the development of IR, cardiovascular events, or other metabolic disruptions, providing potential benefits to health [5]. Therefore, dietary total antioxidant capacity (TAC) is an accepted indicator of diet quality and has emerged as a useful tool to investigate the potential beneficial effects of dietary antioxidants occurring in mixed diets as well as putative synergistic and redox interactions [6].

On the other hand, a number of evidences suggest that chronic consumption of high glycemic index (GI) foods may lead to chronically high OS and IR [7,8]. In this sense, it has been demonstrated that carbohydrate quality plays a significant role in the onset of several chronic diseases, such as diabetes and heart disease [9,10]. An important nutritional feature of carbohydrate-rich foods is the GI, which describes the blood glucose response after sugar consumption [11]. However, GI does not consider the amount of carbohydrates present in foods; therefore, the concept of glycemic load (GL) was introduced [12]. GL is based on the GI and the amount of carbohydrate in the food. GL is calculated by multiplying the grams of available carbohydrate in the food by the food’s GI and then dividing it by 100 [12]. 

The objective of this study was to investigate potential associations between dietary TAC, GI, and GL concerning insulin resistance in subjects with NAFLD who have cardiometabolic risk.

## 2. Results

Descriptive features of the population distributed by tertiles of dietary TAC are reported in Table 1. At baseline, the average age of participants was 50.8 years old of which 42% were women. According to International Diabetes Federation (IDF) criteria [13], 68.5% of subjects suffered from metabolic syndrome and 8.6% suffered from diabetes mellitus. Participants with a higher value of dietary TAC had significantly lower insulin concentration and homeostatic model assessment of insulin resistance (HOMA-IR) as well as less hepatic fat accumulation (*p* < 0.05 for all comparisons) than those with lower values of dietary TAC (Table 1). 

The analysis of the dietary pattern found significant differences among TAC tertiles in consumption of fiber, vitamin C, vitamin E, folic acid, and fermented beverages rich in phenolic compounds (Table 2). There was an increasing trend in energy intake among tertiles of TAC, with subjects in the third tertile having significantly higher values of total energy intake (*p* < 0.001 for all comparisons); therefore, appropriate adjustments were performed to interpret the data. Furthermore, no differences in total carbohydrate consumption among tertiles of TAC were found, but the fiber intake was found to increase throughout the tertiles (Table 2). 

**Figure 1:**
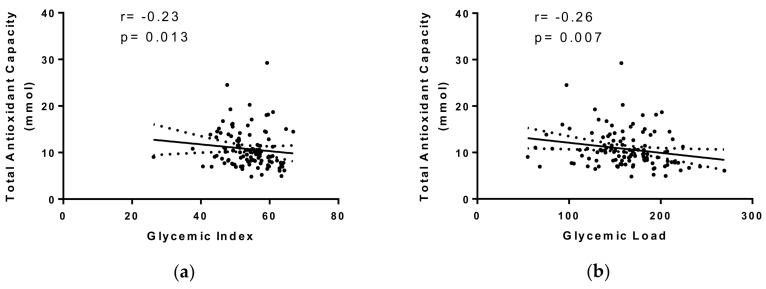
(**a**) Partial correlation between TAC and glycemic index (GI) adjusted by age, sex, BMI, and total energy intake. (**b**) Partial correlations between TAC and glycemic load (GL) adjusted by age, sex, BMI, and total energy intake.

Partial correlation analyses adjusted for age, sex, body mass index (BMI), and daily energy intake evidenced inverse associations between GI (*r* = −0.23, *p* < 0.05) and GL (*r* = −0.26, *p* < 0.05) and TAC (Figure 1).

**Figure 2:**
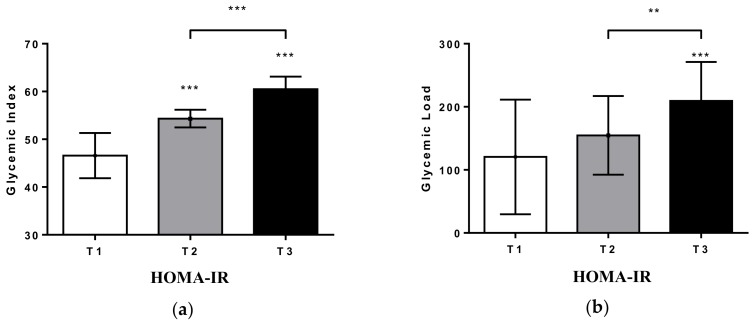
(**a**) Description of GI according to tertiles of HOMA-IR. (**b**) Description of GL according to tertiles of HOMA-IR. Significance is considered ** *p* < 0.01, *** *p* < 0.001.

Participants were also classified according to tertiles of HOMA-IR, and the diet quality was explored. Subjects with higher values of HOMA-IR had a significantly higher GI as well as a higher GL (*p* < 0.001 for all comparisons) than those with lower values of HOMA-IR, as shown in Figure 2. However, the significance disappeared after adjustment by total energy intake (GI: T1= 53.6 (2), T2 = 53.6 (1), T3 = 54.0 (1), *p* = 0.319; GL: T1 = 151.6 (95), T2 = 156.7 (59), T3 = 175.4 (51), *p* = 0.319).

Finally, a linear regression analysis was carried out to assess the impact of all these dietary variables on insulin resistance (HOMA-IR) as showed in Table 3. Some variables associated with HOMA-IR were TAC (β= −1.33 (2.63; −0.04); *p* = 0.044), GI (β = 0.12 (0.03; 0.21); *p* = 0.044), and GL (β = 0.02 (0.001; 0.03); *p* = 0.044). After multivariate adjustment, the final model showed that GI, GL, and TAC were able to explain 28.49% (*p* < 0.001) of the variability of HOMA-IR.

Complementarily, a sensitivity analysis involving the main outcomes was carried out by excluding diabetic participants. A total of 10 diabetics were excluded. When variables were assessed among TAC tertiles, some minor differences were observed. Participants in the third TAC tertile were found to have less hepatic fat and lower circulating insulin concentrations. Differences in HOMA-IR among TAC tertiles were marginally significant when excluding diabetic participants, but the same statistical tendencies were maintained (T1 = 5.6 (7); T2 = 6.1 (5); T3 =3.6 (2)). When all of these variables were included in a multivariate model excluding diabetic subjects, approximately 23.5% of the variability of the HOMA-IR was explained by these variables (*p* model < 0.001).

## 3. Discussion

The main result obtained in this work was that dietary TAC was associated with insulin resistance. Participants that reported higher dietary TAC showed lower insulin and HOMA-IR concentrations. Moreover, other important nutritional factors, such as GI and GL, were also related to the dietary TAC and insulin resistance. 

According to the baseline characteristics of the study participants, it is important to specify that we included subjects that had been diagnosed with NAFLD. Additionally, 8% of these subjects were non-insulin-dependent diabetics treated with metformin (stable dose) [14]. Taking into consideration that glucose metabolism is different between diabetic and nondiabetic participants, a sensitivity analysis was carried out excluding diabetics. Although some minor differences were found, hepatic fat, GL, and GI were associated to HOMA-IR in both samples (including or not including diabetics). In addition, TAC appeared to have a protective role in both NAFLD and type 2 diabetes. On the one hand, TAC had beneficial effects on glucose tolerance and regulation of insulin sensitivity in diabetic participants. On the other hand, TAC seemed to have potential useful effects on the prevention of type 2 diabetes (T2DM) and its complications (nondiabetic participants).

It is also important to highlight the essential role of insulin resistance in NAFLD as a key mediator in the development of NAFLD due to its impact on the increase in de novo lipogenesis and dysfunction in the release of free fatty acids (FFAs) and triglycerides from the liver. These risk factors are also associated with the development of T2DM, explaining the high rate of these diseases occurring concomitantly [15].

Dietary factors play a decisive role in OS in the body cells involved in the onset and development of diverse chronic diseases (obesity, diabetes, etc.) as reported elsewhere [16]. In this framework, several studies have evidenced the potential ability of nutritional intake to modify antioxidant status after the consumption of foods [17,18]. 

In this context, scores and diet intake measurements are useful tools to evaluate diet–disease relationships. Thus, dietary TAC is a validated indicator of diet quality and is an appropriate instrument to evaluate the potential properties of dietary antioxidants. Interestingly, TAC of diet has been related to the consumption of some specific food groups, such as vegetables, fruits, and legumes [19], which are an important source of antioxidants. Furthermore, a recent study evidenced that dietary TAC has a positive association with plasma TAC [20,21], suggesting that it may constitute a convenient instrument to determine antioxidant intake, although this outcome was not found in our trial.

This study revealed that individuals with a higher antioxidant intake were found to have lower values of HOMA-IR and insulin. Indeed, increasing evidence supports a role of OS in the aetiology and progression of diabetes, possibly originating through increased free-radical production [22]. Therefore, pancreatic β cells are susceptible to reactive oxygen species. Thus, by damaging mitochondria, OS could induce apoptosis of pancreatic β cells, impair insulin secretion, and dysregulate glucose concentrations [23,24]. Moreover, the potential effects of antioxidants may happen through the modification of lipid and carbohydrate metabolism as well as increased insulin sensitivity [25].

Notably, many scientific articles have highlighted the association of a dietary pattern rich in fruit and vegetables with a lower risk of diabetes [26]. In a recent meta-analysis study, the investigators evidenced the association of estimated antioxidant intake with a 13% reduction in the risk of diabetes, mainly attributed to vitamin E and carotenoids [27]. Apart from antioxidative mechanisms, elevated dietary fiber, folic acid, and magnesium intake are among the factors that could mediate the effect of a healthy dietary pattern to type 2 diabetes [28]. In accordance with the authors of the previous meta-analysis study [25], significant differences among TAC groups in fiber, folic acid, vitamin C, and vitamin E consumption were found in our study. This suggests that these components of diet are responsible for potential effects on human health because they are related to the supply of dietary antioxidants in addition to their role on GI values. Additionally, we found no significant differences in marine n-3 polyunsaturated fatty acids consumption among TAC tertiles. However, it cannot be dismissed that n-3 could have beneficial effects on the TAC and also the GI as there have been numerous evidences proposing that an increased intake of this dietary component could reduce the risk of cardiovascular disease and insulin resistance condition, improving some adverse metabolic features due to its potential antioxidant and antihyperglycemic properties [29].

Once the association of dietary TAC with IR was confirmed, the possible relationships between IR and dietary GI and GL were assessed due to high GI and GL diets having been suggested as promoting the initiation and progression of insulin resistance and OS through the generation of reactive oxygen species produced in the mitochondria in response to glucose surges [30]. Inflammation is another possible mechanism that could explain the mentioned association. Indeed, it has been proved that low-GI carbohydrates and, to a minor extent, low-protein consumption might specifically decrease low-grade inflammation and related comorbidities [31]. Therefore, important associations of HOMA-IR with GI and GL were identified. However, the significance disappeared after adjustment by total energy intake, suggesting that total energy exposure should be considered in the management of IR.

In addition, the genetic background may have some effects on the sensitivity for developing insulin resistance and other metabolic conditions. In this context, it is important to consider some individual variations in the response to specific dietary patterns [32]. Therefore, the genetic background could partially predetermine the body response of food consumption; consequently, TAC, GI, and GL values could be influenced by genetic predisposition.

Moreover, the current study proves that dietary TAC is associated with GI and GL of diet. Thus, our data found that TAC as a measure of OS was negatively associated with dietary GI and GL while simultaneously being associated with IR and OS.

However, the present study has some limitations that must be considered. Firstly, it could be beneficial to increase the number of participants to raise the statistical power and to ensure more robust outcomes. Secondly, as it was a cross-sectional design, causal effects could not be inferred. Thirdly, food frequency questionnaire (FFQ) and dietary scores are known to contain a certain degree of measurement error, which might have affected results that depended on such evaluation. Fourthly, there is a lack of reliable data on GI values for some foods, which may have led to inaccurate values. Finally, it is well known that dietary TAC is a measure that varies according to the geographic location, seasonality, variety analyzed, cultivation methods used, water and sun availability, storage conditions, food processing, and cooking of the examined food group [33]. On the other hand, some strengths can also be mentioned. The selection of participants was carefully carried out. Despite the small sample size, important associations were found. Additionally, the tested research hypothesis that evaluated a holistic dietary approach of diet in regard to the intake of antioxidants and glycemic biomarkers has rarely been investigated in free-living populations. 

Thus, the current findings are of importance from a public health point of view as well as for personalized nutrition as TAC, GI, and GL of diet can specifically prevent and ameliorate insulin resistance manifestations and control glycemic markers, although it cannot totally cure insulin resistance condition in subjects with a cardiometabolic risk profile.

## 4. Materials and Methods 

### 4.1. Subjects

This cross-sectional analysis was conducted with baseline data of a subsample of participants recruited in the fatty liver in obesity (FLiO) Spain study. A total of 112 overweight or obese adults (age: 50.8 ± 9 years old; BMI: 33.9 ± 4 kg/m^2^) were included in the trial. The inclusion criteria to participate in the study were defined as age 40–80 years old, BMI ≥27.5 to <40 kg/m^2^ and suffering from NAFLD (diagnosis made by hepatology professionals using an ultrasonography technique with appropriate equipment as described by the manufacturer (Siemens ACUSON S2000 and S3000, Erlangen, Germany)). Subjects suffering from the following conditions were not included: endocrine disorders (hyperthyroidism or uncontrolled hypothyroidism); known liver disease (other than NAFLD); alcohol abuse (>21 and >14 units of alcohol per week in men and women, respectively (ex 1 unit = 125 mL of wine); pharmacological treatments with immunosuppressants, cytotoxic agents, systemic corticosteroids, or other drugs that could potentially cause hepatic steatosis or alteration of liver tests; presence of active autoimmune diseases or requiring pharmacological treatment; acute infections; weight loss ≥3 kg in the last three months; serious psychiatric disorders; no autonomy, inability to follow the diet including food allergies or intolerances and/or lifestyle recommendations as well as difficulties to follow scheduled visits.

All procedures performed in the study were in accordance with the ethical guidelines of the Declaration of Helsinki and was appropriately registered following such criteria (www.clinicaltrials.gov; NCT03183193). The study protocol and informed consent document were approved by the Research Ethics Committee of the University of Navarra on 24 April 2015 (ref. 54/2015). A written informed acceptance was obtained from all participants.

### 4.2. Anthropometric and Biochemical Measurements

Anthropometric measurements (body weight, waist circumference) were determined in fasting conditions following previously described standardized procedures [34]. The BMI was calculated as body weight divided by squared height (kg/m^2^). The body composition was analyzed by dual-energy X-ray absorptiometry (DXA) according to the manufacturer′s instructions (Lunar idxa, encore 14.5, Madison, WI, USA), and hepatic measurements (fat content by Dixon) were assessed by magnetic resonance imaging (MRI) (Siemens 1.5 T). 

Blood pressure was determined using an automatic monitor device following World Health Organization criteria (Intelli Sense. M6, OMRON Healthcare, Hoofddorp, The Netherlands). Blood glucose, glycosidic hemoglobin (HbA1c), triglycerides (TG), total cholesterol (TC), high-density lipoprotein (HDL-c), homocysteine, alanine aminotransferase (ALT), and aspartate aminotransferase (AST) were measured on a suitable autoanalyzer (Pentra C-200; HORIBA ABX, Madrid, Spain) with specific kits and using standardized methods.

Insulin and C-reactive protein (CRP) values were quantified with specific ELISA kits (Demeditec, Kiel-Wellsee, Germany) in a Triturus auto-analyzer (Grifols, Barcelona, Spain). IR was estimated using HOMA-IR, which was calculated using the following formula [35]: HOMA-IR = (insulin (µU/mL) × glucose (mmol/L))/22.5. The low-density lipoprotein (LDL-c) levels were calculated using the Friedewald formula [36]: LDL-c = TC − HDL-c − TG/5.

### 4.3. Dietary Data

Dietary intake was assessed at baseline with a validated 137-item semiquantitative FFQ [37]. Each item in the questionnaire included a typical portion size. For each food item, daily food consumption was estimated after multiplying the portion size by the consumption frequency as described elsewhere. Nutrient composition of the food items was derived from accepted Spanish food composition tables [38].

The dietary TAC score was calculated by computing the individual TAC values from the ferric reducing antioxidant power assay of each food as previously reported [39,40,41] and was expressed in mmol/100 g of food. The mean TAC value of the foods contained in each item was used to calculate the dietary TAC score from the FFQ [42].

GI values for single food items on the food frequency questionnaire were derived from the International Tables of Glycemic Index and Glycemic Load Values: 2008 and a website created and maintained by the University of Sydney (Australia) [43,44]. Total dietary GI was estimated by multiplying the amount of available carbohydrate (g) of each food item by its GI. Then, the sum of these products was divided by the total carbohydrate intake. As the amount of carbohydrate in an overall diet can vary, we also applied the concept of GL, which represents the amount of carbohydrates multiplied by the average GI and divided by 100 [45].

### 4.4. Statistical Analysis

The data was expressed as a mean and standard deviation. Normality of the analyzed variables was determined according to the Shapiro–Wilk test. Measured variables were categorized by tertiles of TAC (T1 (<8.6 mmol) vs. T2 (8.6–11.36 mmol) vs. T3 (>11.36 mmol). Differences between groups (T1, T2, and T3) were assessed by the ANOVA test with a correction for multiple comparisons by following the Bonferroni adjustment (post hoc test) concerning quantitative variables and the chi-square test or Fisher’s exact test were applied concerning categorical variables.

Partial correlations adjusted for age, sex, BMI, and total energy intake were performed to further explain the association of GI and GL with TAC. Variables associated with insulin resistance (HOMA-IR) were selected to be included in a linear regression model. Thus, a multivariable linear regression analysis was set up to assess the potential influence of independent variables such as TAC, GI, GL, and hepatic fat by MRI in insulin resistance after adjustment for potential confounders (sex, age, and total energy intake) when indicated. Confidence intervals were used to describe the lineal regression coefficient (β) values. 

Statistical analysis and graphs were performed using STATA 12 software for Windows (StataCorp, College Station, TX, USA). All *p*-values were two-tailed. Values of *p* < 0.05 were considered to be statistically significant.

## 5. Conclusions

The results from the current study reinforces the concept that dietary TAC, GI, and GL are associated with insulin resistance and metabolic outcomes and are potential markers of diet quality for targeted precision nutrition. This work suggests an association between dietary TAC and GI and GL. These scientific insights suggest that a well-designed precision nutritional therapy that promotes the TAC, GI, and GL of diet could specifically prevent and ameliorate insulin resistance manifestations and control glycemic markers in addition to obesity and cardiovascular disease.

## Figures and Tables

**Table 1:** Descriptive characteristics of the study participants according to tertiles of total antioxidant capacity (TAC).

*n* = 112	All Participants	T1 (<8.6 mmol) (*n* = 38)	T2 (8.6–11.36 mmol) (*n* = 37)	T3 (>11.36 mmol) (*n* = 37)	*p*-Value
Sex (men/women)	65/47	22/16	22/15	20/17	0.863
Age (years)	50.8 (9)	48.1 (10)	54.2 (9) #	50.3 (8)	0.017
BMI (kg/m^2^)	33.9 (4)	33.6 (4)	34.6 (4)	33.1 (3)	0.241
**Cardiometabolic risk factors**					
Waist circumference (cm)	109.8 (8)	109.4 (11)	111.6 (10)	108.6 (9)	0.400
Total fat mass (%)	42.9 (6)	43.7 (6)	43.0 (7)	42.8 (6)	0.674
Visceral fat mass (g)	2374 (1051)	2530 (1293)	2371 (873)	2211 (1010)	0.455
Hepatic fat by MRI (%)	9.4 (9)	10.75 (13)	11.5 (9) †	5.7 (4.8)	0.023
Blood pressure levels (mmHg)					
Systolic	131 (17)	130 (14)	131 (15)	130 (21)	0.984
Diastolic	87 (10)	86 (10)	87 (7)	88 (10)	0.521
Diabetes mellitus (%)	8.6	8.1	10.8	8.1	0.898
Metabolic syndrome (%)	68.5	67.6	70.3	70.3	0.960
Glucose (mg/dL)	108.0 (30)	114.2 (45)	112.7 (25)	99.3 (15)	0.081
HbA1c (%)	5.9 (11)	6.2 (2)	5.9 (1)	5.7 (0.5)	0.134
Insulin (U/L)	19.0 (12)	18.1 (10)	22.3 (14)	14.8 (7) *	0.010
HOMA-IR	5.4 (5)	5.7 (6)	6.5 (5)	3.6 (2) *	0.031
TG (mg/dL)	137.0 (77)	137.9 (91)	139.1 (80)	137.6 (69)	0.996
TC (mg/dL)	195.3 (38)	193.2 (36)	193.7 (42.4)	203.0 (39.2)	0.485
LDL-c/HDL-c ratio	2.4 (1)	2.4 (1)	2.4 (0.8)	2.4 (0.9)	0.965
TyG index	1.3 (0.7)	1.2 (0.6)	1.3 (0.7)	1.4 (0.8)	0.404
Homocysteine (µmol/L)	15.6 (6)	15.6 (8)	15.8 (5)	14.9 (5)	0.790
CRP (mg/dL)	0.5 (1.2)	0.5 (0.8)	0.4 (0.5)	0.7 (2.1)	0.739
AST/ALT ratio	0.8	0.8 (0.3)	0.8 (0.2)	0.86 (0.3)	0.406
Cholesterol-lowering drugs (no/yes)	95/17	30/7	30/7	34/3	0.329
Blood pressure medications (no/yes)	86/26	28/9	29/8	29/8	0.950
**Lifestyle factors**					
Smoking habit (*n*)					0.946
Never	32	11	9	11	0.966
Former smoker	43	15	13	15	0.946
Sporadically	5	2	2	1	0.871
Current smoker	19	5	5	9	0.871
Physical activity (*n*)					0.579
Never	46	17	15	14	0.417
Mild	26	9	6	10	0.579
Moderated	26	5	11	10	0.669
Elevated	14	6	5	3	0.508

Values are represented as Mean (SD). Abbreviations: BMI: body mass index; MRI: magnetic resonance imaging; HbA1c: glycosidic hemoglobin; HOMA-IR: homeostatic model assessment of insulin resistance; TG: triglycerides; TC: total cholesterol; LDL-c: low-density lipoprotein cholesterol; HDL-c: high-density lipoprotein cholesterol; TyG index: triglyceride-glucose index; CRP: C-reactive protein; AST: aspartate aminotransferase; ALT: alanine aminotransferase. * *p* was significant between participants with TAC < 8.6 mmol and TAC > 11.36 mmol. # *p* was significant between participants with TAC <8.6 mmol and TAC 8.6–11.36 mmol. † *p* was significant between participants with TAC 8.6–11.36 mmol and TAC > 11.36 mmol.

**Table 2:** Description of the nutrient and food consumption according to tertiles of TAC.

*n* = 112	All	T1 (<8.6 mmol) (*n* = 38)	T2 (8.6–11.36) (*n* = 37)	T3 (>11.36) (*n* = 37)	*p*-Value
**Energy and macronutrients**					
Energy intake (kcal/day)	2691 (1010)	2210 (570)	2701 (744)	3169 (1343) *	<0.001
Carbohydrates (%E)	43.0 (7)	42.6 (7)	44.3 (7)	42.2 (8)	0.405
Dietary fiber (g/day)	24.8 (9)	20.3 (6)	26.4 (18) #	27.1 (9) *	<0.001
Total protein (%E)	17.4 (4)	18.1 (4)	17.7 (4)	16.5 (3)	0.140
Total lipid (%E)	37.1 (7)	38.1 (7)	36.4 (7)	36.9 (7)	0.558
**Micronutrients**					
Vitamin A (µg/day)	1100.2 (820)	908.9 (531)	1089.3 (627)	1309.3 (1151)	0.108
Vitamin C (mg/day)	200.8 (115)	157.9 (68.6)	198.7 (84)	245.3 (159) *	0.004
Vitamin D (µg/day)	6.2 (4)	5.5 (3)	6.2 (4)	6.8 (4)	0.306
Vitamin E (mg/day)	10.5 (4)	8.9 (3)	10.6 (4)	12.0 (5) *	0.009
Folic acid (µg/day)	362.8 (140)	290.4 (75)	389.8 (159)	408.9 (147) *	<0.001
Phenolic compounds rich fermented beverages (g)	8.7 (11)	3.8 (5)	6.1 (7)	16.7 (14) *	<0.001
Marine Ω3 (g/day)	0.64 (0.4)	0.62 (0.4)	0.62 (0.4)	0.69 (0.3)	0.611

Values are represented as Mean (SD). * *p* was significant between participants with TAC < 8.6 mmol and TAC > 11.36 mmol. # *p* was significant between participants with TAC < 8.6 mmol and TAC 8.6–11.36 mmol.

**Table 3:** Multiple linear regression models with the HOMA-IR as the dependent variable and using biochemical, hepatic, and dietetic factors as independent variables.

	Model ^1^	
Variables	β (95% CI)	*p*-Value
GI	0.12 (0.03; 0.21)	0.012
GL	0.02 (0.001; 0.03)	0.037
TAC (mmol/day)		
TAC < p50	1	
TAC ≥ p50	−1.33 (2.63; −0.04)	0.044

^1^ Model: sex, age, total energy intake, and hepatic fat adjusted. Abbreviations: p50: 50th percentile.

## Abbreviations

TAC	total antioxidant capacity
GI	glycemic index
GL	glycemic load
IR	insulin resistance
FFQ	food frequency questionnaire
DXA	dual-energy X-ray absorptiometry
CRP	C-reactive protein
MRI	magnetic resonance imaging
OS	oxidative stress
NAFLD	nonalcoholic fatty liver disease
BMI	body mass index
HbA1c	glycosidic hemoglobin
TG	triglycerides
TC	total cholesterol
LDL-c	high-density lipoprotein
HDL-c	low-density lipoprotein
AST	aspartate aminotransferase
ALT	alanine aminotransferase
FLiO	fatty liver in obesity—Spain
